# Kinesin family member 2A links with advanced tumor stage, reduced chemosensitivity and worse prognosis in gastric cancer

**DOI:** 10.1002/jcla.24313

**Published:** 2022-03-21

**Authors:** Fei Bai, Zhuo He, Huijun Zhou, Wei Gan

**Affiliations:** ^1^ Department of Gastroduodenal Pancreas Surgery Hunan Cancer Hospital & The Affiliated Hospital of Xiangya School of Medicine Central South University Changsha China; ^2^ Department of Gastroenterology and Urology Hunan Cancer Hospital & The Affiliated Hospital of Xiangya School of Medicine Central South University Changsha China; ^3^ Department of General Surgery The Second Xiangya Hospital Central South University Changsha China

**Keywords:** chemosensitivity, clinicopathological features, gastric cancer, Kinesin family member 2A, survival

## Abstract

**Background:**

Kinesin family member 2A (KIF2A) induces gastric cancer (GC) growth and invasion, while its clinical relevance in GC patients is not reported. This study aimed to investigate the linkage of KIF2A with clinicopathological features, prognosis, and chemosensitivity of GC.

**Methods:**

A total of 160 surgical GC patients were reviewed, with their tumor and adjacent tissues acquired for immunohistochemical (IHC) assay to measure KIF2A expression, then scored by a semi‐quantitative method (IHC score: 0–12). KIF2A siRNA or nonsense‐siRNA were transfected into HGC‐27 and NCI‐N87 cells underwent various concentrations of capecitabine or oxaliplatin treatment followed by chemosensitivity assessment.

**Results:**

Kinesin family member 2A expression was elevated in the tumor tissue compared to the adjacent tissue (IHC score: 5.6 ± 3.1 vs. 2.9 ± 1.7, *p* < 0.001). Besides, tumor KIF2A expression was related to larger tumor size (*p* = 0.014), higher N stage (*p* = 0.004) and TNM stage (*p* = 0.011); however, it was not linked with other clinicopathological features (all *p *> 0.05). Signally, tumor KIF2A high expression predicted poor overall survival (*p* = 0.037). After adjustment via multivariate Cox's regression, tumor KIF2A high expression independently linked with worse disease‐free survival (*p* = 0.033). Finally, KIF2A knockdown improved the oxaliplatin chemosensitivity vastly but only slightly affected capecitabine chemosensitivity in HGC‐27 and NCI‐N87 cells.

**Conclusion:**

Kinesin family member 2A reflects larger tumor size, advanced TNM stage, improved chemosensitivity, and predicts unfavorable survival in GC.

## Graphical abstract

This study aimed to explore the linkage of KIF2A with clinicopathological features and prognosis, then investigate its effect on the chemosensitivity in gastric cancer. The results showed that KIF2A expression was increased in the tumor tissue than in the adjacent tissue. Besides, tumor KIF2A expression was correlated with larger tumor size, higher N stage and TNM stage. Moreover, tumor KIF2A high expression was associated with poor overall survival; meanwhile, KIF2A expression (high vs. low) independently associated with shorter DFS. Furthermore, KIF2A knockdown improved the chemosensitivity to oxaliplatin vastly and only increased that to capecitabine mildly in HGC‐27 and NCI‐N87 cells.

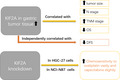

## 1 INTRODUCTION

Gastric cancer (GC) is the fifth most common and the third most lethal cancer globally, with approximately 950,000 cases of diagnosis and over 780,000 deaths in 2018.^1^, ^2^, ^3^ Meanwhile, changes in diet and lifestyle, increased use of salt and alcohol, *Helicobacter pylori* (*H*. *pylori*) infection elevated the risk of GC in recent decades.^4^ According to different disease conditions, several therapeutic modalities are recommended: for non‐metastatic GC patients, on the basis of their disease status, surgical resection only, surgical resection followed by adjuvant therapy or neoadjuvant therapy followed by surgical resection bring a relatively favorable survival outcome^5^; otherwise, for those with metastatic GC, systemic chemotherapy and/or targeted therapy remains the only treatment choice.^6^ With the advancement of treatment modalities, the 5‐year survival rate of non‐metastatic GC is improved; however, the general survival of GC is still unsatisfying.^7^, ^8^ Meanwhile, GC patients would possibly encounter a high recurrence risk and die of disease relapse even accepting successful surgical resection.^9^, ^10^ Therefore, finding out new biomarkers to identify GC patients who are easier to relapse and possess high mortality risk is essential.

Kinesin family member 2A (KIF2A), an M‐type nonmotile microtubule depolymerase, plays vital roles in the progression of various tumors.^11^, ^12^, ^13^, ^14^, ^15^ For instance, via activating phosphatidylinositol 3‐kinase/protein kinase B (PI3K/AKT) and mitogen‐activated protein kinases/extracellular signal‐regulated kinase (MAPK/ERK) signaling pathways, KIF2A can facilitate lung adenocarcinoma cell proliferation and suppress apoptosis^13^; by means of suppressing microRNA (miR)‐206, KIF2A promotes ovarian cancer cell proliferation and migration^14^; meanwhile, KIF2A also contributes to osteosarcoma cell (MG‐63 and U2OS cells) proliferation, migration and invasion.^15^ Moreover, referring to GC, previous studies suggest that KIF2A promotes the proliferation of GC cells via the AKT signaling pathway, thus participating in the progression of GC.^16^ Clinically, KIF2A could serve as a potential prognostic biomarker for facilitate prognostication of lung adenocarcinoma and esophageal squamous cell carcinoma patients.^13^, ^17^ Based on the above‐mentioned information, we hypothesized that KIF2A might be a potential biomarker for GC. However, few previous studies had been performed.

The present study aimed to detect the KIF2A expression in GC tissue, and explore its correlation with clinical characteristics and prognosis in GC patients, then assess its effect on chemosensitivity in GC cell lines.

## 2 MATERIALS AND METHODS

### 2.1 Patients

The medical records of GC patients who underwent surgical resection in our hospital from January 2015 to December 2019 were reviewed. A total of 160 GC patients were screened out and eligible for study analysis, based on the following criteria: (1) had a clinicopathological diagnosis of primary GC; (2) underwent surgical resection; (3) had complete clinicopathological data and survival data; (4) had accessible formalin‐fixed and paraffin‐embedded (FFPE) specimens of tumor and adjacent tissues; (5) had no history of other cancers or malignant diseases at diagnosis. The exclusion criteria were: (1) age less than 18 years old; (2) patients’ specimens were not available for immunohistochemistry assay; (3) lost to follow‐up within 6 months after surgery. Approval was acquired from the Institutional Review Board of our hospital with the written informed consent omitted.

### 2.2 Data collection

By reviewing the medical records, the following data were extracted: (1) clinical characteristics: age, gender, smoke and drink status, comorbidities, *H. pylori* infection status, tumor location, tumor differentiation, tumor size, T stage, N stage as well as Tumor, Node and Metastasis (TNM) stage; (2) adjuvant treatment: adjuvant chemotherapy and radiotherapy (patients with TNM stage II–III received XELOX (capecitabine and oxaliplatin) regimen, while some patients stopped the regimen due to intolerable toxicity; meanwhile, some patients with TNM stage III also received radiotherapy concurrently); (3) survival data: date of surgery, date of disease relapse, progression or death, and the date of last follow‐up (for estimation of disease‐free survival (DFS) and overall survival (OS)). At the time of study analysis, the survival data were updated to March 2021. The median follow‐up duration was 46.5 months (calculated by reverse Kaplan–Meier method) with a range from 12.5 to 71.4 months.

### 2.3 Immunohistochemistry (IHC) staining

The FFPE specimens were processed by immunohistochemical staining for semiquantitative analysis of KIF2A expression. The IHC staining was implemented according to the standard procedures as described in a previous study.^14^ The following antibodies were respectively applied as primary and secondary antibodies: Rabbit polyclonal to KIF2A at a dilution ratio of 1/300 (ab197988, Abcam), and Goat Anti‐Rabbit IgG H&L (HRP) at a dilution ratio of 1/50,000 (ab6721, Abcam). Diaminobenzidine (Invitrogen) was used as chromogenic reagent and hematoxylin (Sigma‐Aldric) was applied for counterstaining. The IHC staining results were assessed by two pathologists independently. The staining intensity and the percentage of positive cells were respectively scored from 0 to 3 and from 0 to 4, referring to a previously published methodology.^18^ An IHC score was yielded by multiplying these two scores, and the mean score of two pathologists was used as final result in the analysis. The KIF2A expression was categorized as high (IHC score >3) and low (IHC score ≤3).^18^

### 2.4 Cell culture

Two human GC cell lines HGC‐27 and NCI‐N87 (Cell Blank of Chinese Academy of Sciences) were used for in vitro experiment. The RPMI1640 medium (Gibco) and 10% fetal bovine serum (Gibco) were applied for cell culture, which was performed in the following conditions: air 95%, carbon dioxide 5%, and 37°C.

### 2.5 Determination of chemosensitivity after transfection

The siRNAs for KIF2A and nonsense‐siRNA were purchased from the RIBOBIO Biotechnology Co., Ltd, which were respectively transfected into cells by Lipofectamine™ 2,000 Transfection Reagent (Invitrogen). According to the transfection, the cells were termed as follows: (1) KD‐KIF2A (knockdown KIF2A) group: the cells were transfected with siRNAs for KIF2A; (2) KD‐NC (negative control) group: the cells were transfected with nonsense‐siRNA; (3) blank group: the cells were not transfected. At 24 h after completion of transfection, all cells (KD‐KIF2A, KD‐NC, blank) were treated with capecitabine (Sigma‐Aldrich) and oxaliplatin (Sigma‐Aldrich), respectively. The concentrations of chemotherapeutic agents were set partly according to a previous study^19^ with some modification as follows: capecitabine, 0, 100, 200, 400, 800, 1600, and 3200 μg/ml; oxaliplatin, 0, 0.2, 0.4, 0.8, 1.6, 3.2, and 6.4 μM. For the determination of drug sensitivity of cells, the relative cell viability at each drug concentration was measured after treatment for 48 h, with the use of Cell Counting Kit‐8 (Beyotime) according to the instruction of the kit.

### 2.6 Statistical analysis

Paired *t* test and McNemar's test were applied for the paired sample comparison. The student's *t* test was used for independent sample comparison. Spearman rank correlation test was used for association analysis. Kaplan–Meier method and log‐rank test were used for DFS and OS analysis. Univariate and multivariate Cox's proportional hazards regression analyses were used to estimate the factors related to DFS and OS. Statistical analysis was completed using SPSS 22.0 (IBM Corp.), and graphs were constructed using GraphPad Prism 7.01 (GraphPad Software Inc.). *p* value < 0.05 indicated statistical significance.

## 3 RESULTS

### 3.1 Clinical characteristics

A total of 160 GC patients were admitted into this study, of which detailed clinical characteristics were shown in Table 1. Briefly, the mean age of GC patients was 58.5 ± 11.1 years. There were 95 (59.4%) male patients and 65 (40.6%) female patients. The numbers of patients with current smoking, current drinking, hypertension, hyperlipidemia, diabetes, and *H. pylori* infection were 46 (28.7%), 61 (38.1%), 37 (23.1%), 45 (28.1%), 24 (15.0%), and 56 (35.0%), respectively. Regarding tumor location, 45 (28.1%), 19 (11.9%), and 96 (60.0%) patients’ gastric tumor located at the cardia, the gastric body and the gastric antrum, respectively. Besides, 20 (12.5%), 116 (72.5%), and 24 (15.0%) patients had well, moderate, and poor tumor differentiation, respectively. Additionally, 40 (25.0%), 68 (42.5%), and 52 (32.5%) patients presented with TNM stage I, II, and III, respectively. Moreover, there were 115 (71.9%) patients received adjuvant chemotherapy and 11 (6.9%) patients received adjuvant radiotherapy.

**TABLE 1:** Clinical characteristics of patients with gastric cancer

Items	Patients (*N* = 160)
Age (years), mean ± SD	58.5 ± 11.1
Gender, no. (%)	
Male	95 (59.4)
Female	65 (40.6)
Current smoke, no. (%)	46 (28.7)
Current drink, no. (%)	61 (38.1)
Hypertension, no. (%)	37 (23.1)
Hyperlipidemia, no. (%)	45 (28.1)
Diabetes, no. (%)	24 (15.0)
*H. pylori* infection positive, no. (%)	56 (35.0)
Tumor location, no. (%)	
Cardia	45 (28.1)
Gastric body	19 (11.9)
Gastric antrum	96 (60.0)
Tumor differentiation, no. (%)	
Well	20 (12.5)
Moderate	116 (72.5)
Poor	24 (15.0)
Tumor size (cm), mean ± SD	3.8 ± 1.2
T stage	
T1	13 (8.1)
T2	35 (21.9)
T3	111 (69.4)
T4	1 (0.6)
N stage	
N0	100 (62.5)
N1	30 (18.8)
N2	25 (15.6)
N3	5 (3.1)
TNM stage	
I	40 (25.0)
II	68 (42.5)
III	52 (32.5)
Adjuvant chemotherapy, no. (%)	115 (71.9)
Adjuvant radiotherapy, no. (%)	11 (6.9)

Abbreviations: *H. pylori*, *Helicobacter pylori*; SD, standard deviation.

### 3.2 KIF2A expression in the tumor tissue and adjacent tissue

**FIGURE 1:**
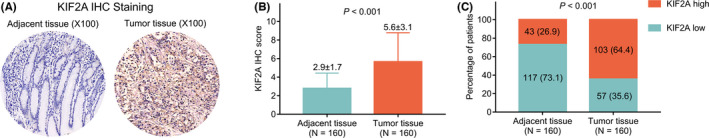
KIF2A expression in GC patients. Examples of KIF2A expression by IHC assay (A); KIF2A IHC score (B); the percentage of KIF2A high and KIF2A low (C) in the tumor tissue and adjacent tissue of GC patients. GC, gastric cancer; IHC, immunohistochemistry; KIF2A, kinesin family member 2A

Kinesin family member 2A expression was detected by IHC assay in tumor and adjacent tissue (Figure 1A). Meanwhile, KIF2A IHC score was increased in the tumor tissue (5.6 ± 3.1) than in the adjacent tissue (2.9 ± 1.7) (*p* < 0.001) (Figure 1B); KIF2A high percentage (defined as KIF2A IHC score >3) was also higher in tumor tissue than in adjacent tissue (*p* < 0.001) (Figure 1C).

### 3.3 Association of KIF2A in tumor tissue with clinical features

**FIGURE 2:**
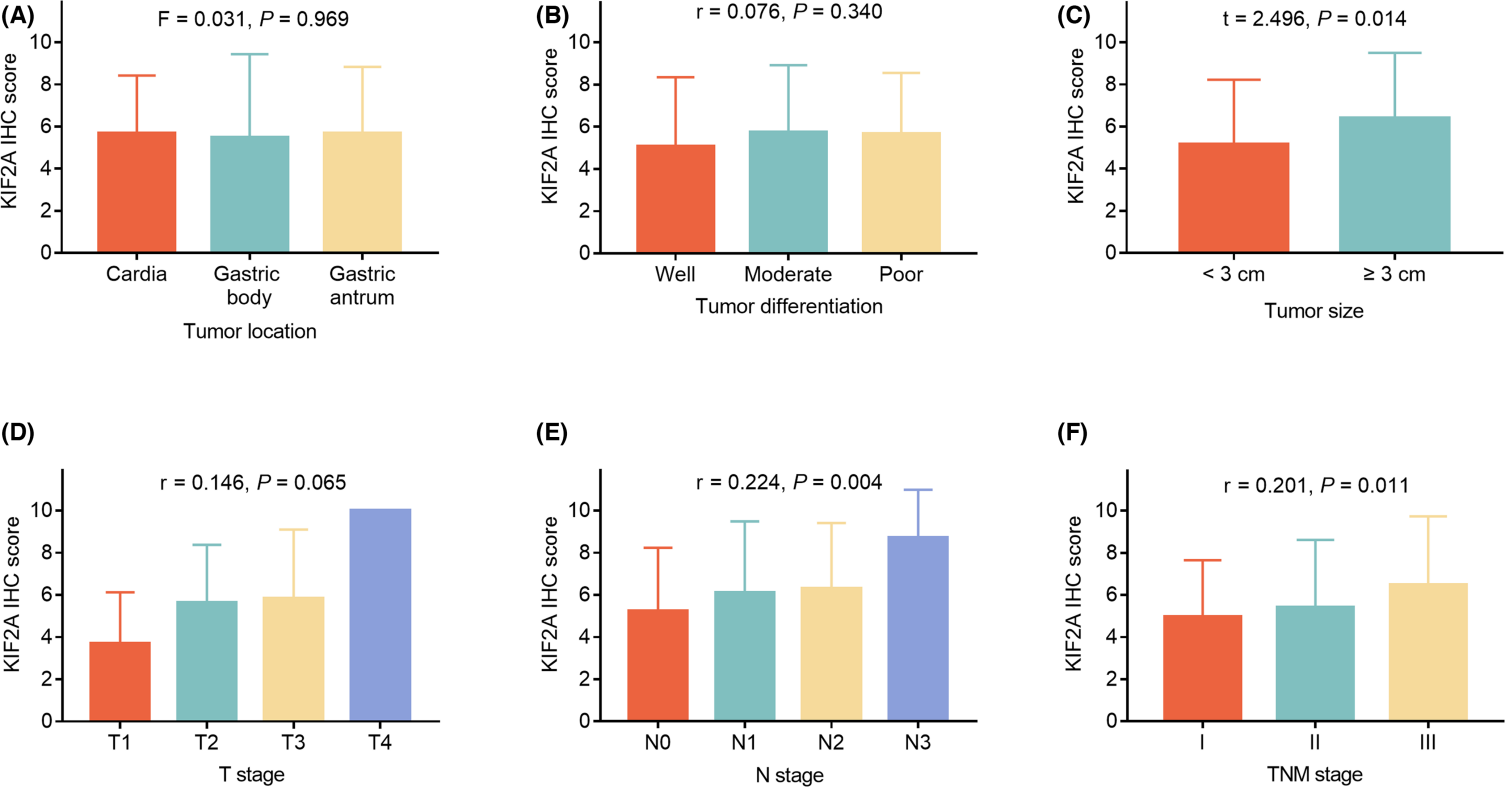
Correlation of KIF2A expression with tumor features in GC patients. Correlation of KIF2A expression with tumor location (A), tumor differentiation (B), tumor size (C), T stage (D), N stage (E), and TNM stage (F). GC, gastric cancer; IHC, immunohistochemistry; KIF2A, kinesin family member 2A; N stage, node stage; T stage, tumor stage; TNM stage, tumor, node and metastasis stage

Elevated KIF2A IHC score was correlated with tumor size≥3 cm (*p* = 0.014), more advanced N stage (*p* = 0.004) and TNM stage (*p* = 0.011). However, no association of KIF2A IHC score with tumor location, tumor differentiation or T stage was found (all *p *> 0.05) (Figure 2A–F).

### 3.4 Correlation of tumor KIF2A with accumulating DFS and OS

**FIGURE 3:**
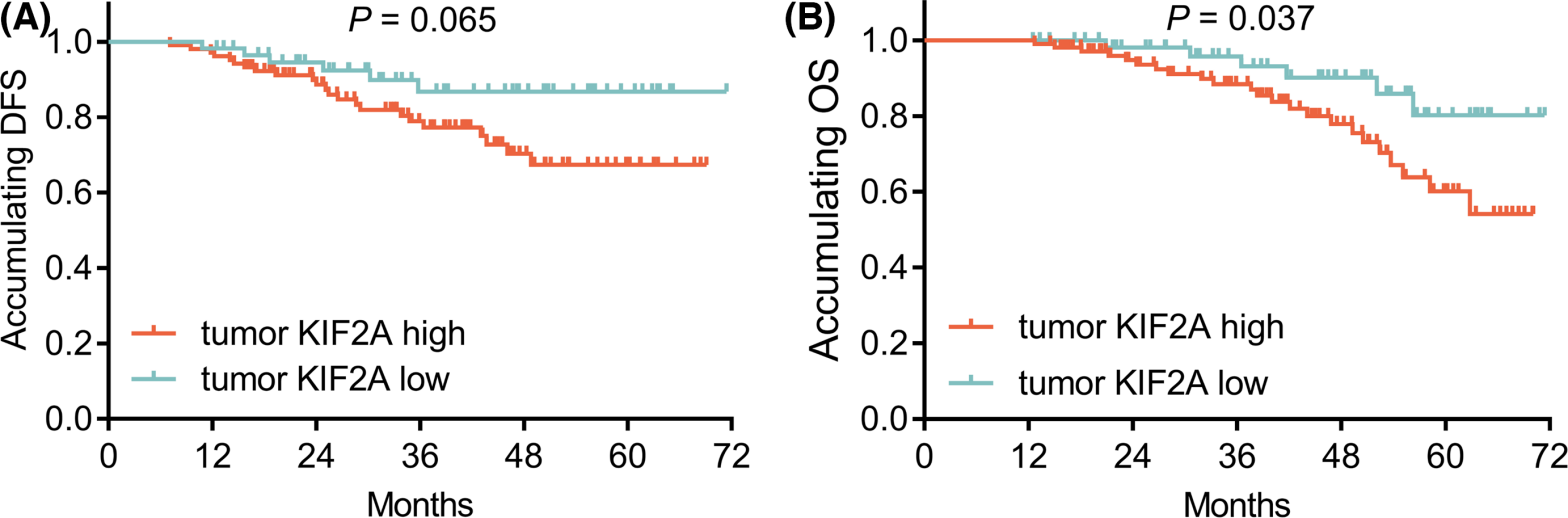
Association of KIF2A expression in tumor with prognosis in GC patients. Association of KIF2A expression in tumor with accumulating DFS (A) and OS (B). DFS, disease‐free survival; GC, gastric cancer; KIF2A, kinesin family member 2A; OS, overall survival

Tumor KIF2A high expression was associated with poor accumulating OS (*p* = 0.037). However, no correlation was found in tumor KIF2A expression with accumulating DFS (*p* = 0.065) (Figure 3A,B).

Regarding DFS, multivariate Cox's regression analysis presented that KIF2A expression (high vs. low) (*p* = 0.033, HR (95% CI): 1.943 (1.057–3.573)), gender (male vs. female) (*p* = 0.002, HR (95% CI): 3.418 (1.566–7.459)), tumor location of cardia (vs. location of antrum) (*p* = 0.044, HR (95% CI): 2.253 (1.023–4.961)), poor tumor differentiation (*p* = 0.001, HR (95% CI): 2.703 (1.490–4.904)), higher TNM stage (*p* < 0.001, HR (95% CI): 3.712 (2.009–6.859)) were all independently associated with unsatisfying DFS, while adjuvant chemotherapy (yes vs. no) (*p* < 0.001, HR (95% CI): 0.078 (0.022–0.270)) was independently associated with longer DFS (Table 2).

**TABLE 2:** Factors affecting DFS

Items	Cox's proportional hazards regression model			
	*p* Value	HR	95% CI	
			Lower	Upper
Univariate Cox's regression				
KIF2A expression (high vs. low)	0.069	1.737	0.958	3.150
Age (≥60 years vs. <60 years)	0.543	0.848	0.499	1.442
Gender (male vs. female)	0.011	2.208	1.202	4.056
Current smoke (yes vs. no)	0.288	0.719	0.390	1.322
Current drink (yes vs. no)	0.556	0.848	0.489	1.470
Hypertension (yes vs. no)	0.277	1.374	0.775	2.436
Hyperlipidemia (yes vs. no)	0.283	0.704	0.371	1.336
Diabetes (yes vs. no)	0.449	1.321	0.643	2.716
*H. pylori* infection positive (yes vs. no)	0.710	0.900	0.516	1.570
Tumor location				
Gastric antrum	Ref.	‐	‐	‐
Gastric body	0.107	0.587	0.308	1.121
Cardia	0.263	1.530	0.727	3.221
Poor tumor differentiation	0.001	2.611	1.503	4.536
Tumor size (≥3 cm vs. <3 cm)	0.497	1.203	0.706	2.051
Higher T stage	0.269	1.344	0.796	2.269
Higher N stage	0.001	1.601	1.216	2.109
Higher TNM stage	0.004	1.742	1.190	2.549
Adjuvant chemotherapy (yes vs. no)	0.608	1.178	0.630	2.200
Adjuvant radiotherapy (yes vs. no)	0.692	1.228	0.444	3.401
Forward stepwise (conditional) multivariate Cox's regression				
KIF2A expression (high vs. low)	0.033	1.943	1.057	3.573
Gender (male vs. female)	0.002	3.418	1.566	7.459
Tumor location				
Gastric antrum	Ref.	‐	‐	‐
Gastric body	0.244	0.669	0.341	1.316
Cardia	0.044	2.253	1.023	4.961
Poor tumor differentiation	0.001	2.703	1.490	4.904
Higher TNM stage	<0.001	3.712	2.009	6.859
Adjuvant chemotherapy (yes vs. no)	<0.001	0.078	0.022	0.270

Note

Factors affecting DFS in gastric cancer patients was analyzed by univariate and forward stepwise multivariate logistic regression model.

Abbreviations: CI, confidence interval; DFS, disease‐free survival; *H. pylori*, *Helicobacter pylori*; HR, hazard ratio.

In terms of OS, multivariate Cox's regression analysis showed that poor tumor differentiation (*p* = 0.043, HR (95% CI): 2.576 (1.029–6.447)), higher TNM stage (*p* < 0.001, HR (95% CI): 7.159 (3.039–16.862)) were independently associated with shorter OS, while adjuvant chemotherapy (yes vs. no) (*p* < 0.001, HR (95% CI): 0.051 (0.012–0.215)) was independently correlated with better OS (Table 3).

**TABLE 3:** Factors affecting OS

Items	Cox's proportional hazards regression model			
	*p* Value	HR	95% CI	
			Lower	Upper
Univariate Cox's regression				
KIF2A expression (high vs. low)	0.044	2.517	1.023	6.192
Age (≥60 years vs. <60 years)	0.093	0.530	0.253	1.111
Gender (male vs. female)	0.438	1.355	0.629	2.922
Current smoke (yes vs. no)	0.057	0.358	0.124	1.030
Current drink (yes vs. no)	0.170	0.565	0.250	1.276
Hypertension (yes vs. no)	0.909	1.049	0.463	2.375
Hyperlipidemia (yes vs. no)	0.209	0.539	0.206	1.414
Diabetes (yes vs. no)	0.934	1.046	0.362	3.023
*H. pylori* infection positive (yes vs. no)	0.795	0.904	0.420	1.945
Tumor location				
Gastric antrum	Ref	‐	‐	‐
Gastric body	0.037	0.352	0.132	0.941
Cardia	0.946	0.963	0.327	2.839
Poor tumor differentiation	0.045	2.196	1.017	4.742
Tumor size (≥3 cm vs. <3 cm)	0.039	2.185	1.042	4.580
Higher T stage	0.282	1.537	0.703	3.361
Higher N stage	0.001	1.871	1.293	2.708
Higher TNM stage	0.015	1.956	1.138	3.361
Adjuvant chemotherapy (yes vs. no)	0.604	0.812	0.368	1.788
Adjuvant radiotherapy (yes vs. no)	0.364	1.742	0.526	5.767
Forward stepwise (conditional) multivariate Cox's regression				
Poor tumor differentiation	0.043	2.576	1.029	6.447
Higher TNM stage	<0.001	7.159	3.039	16.862
Adjuvant chemotherapy (yes vs. no)	<0.001	0.051	0.012	0.215

Note

Factors affecting OS in gastric cancer patients was analyzed by univariate and forward stepwise multivariate logistic regression model.

Abbreviations: CI, confidence interval; *H. pylori*, *Helicobacter pylori*; HR, hazard ratio; OS, overall survival.

### 3.5 Effect of KIF2A expression on chemosensitivity in gastric cancer cell lines

**FIGURE 4:**
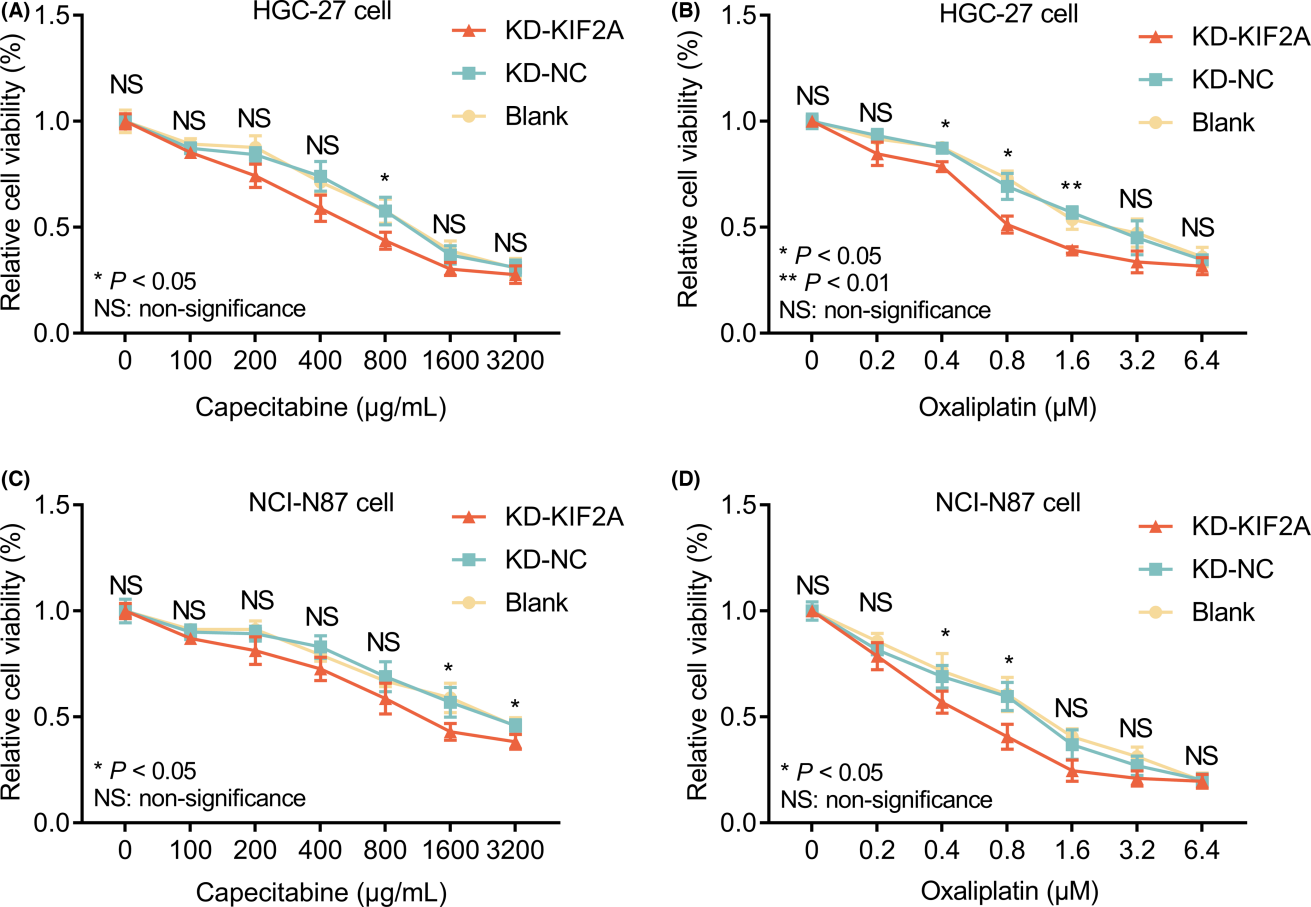
Relative cell viability of HGC‐27 and NCI‐N87 cells after transfection under capecitabine or oxaliplatin treatment. Comparison of relative cell viability of HGC‐27 cells (A) or NCI‐N87 cells (B) between the KD‐KIF2A group and the KD‐NC group under 0, 100, 200, 400, 800, 1600, 3200 μg/ml capecitabine treatment; comparison of relative cell viability of HGC‐27 cells (C) or NCI‐N87 cells (D) between the KD‐KIF2A group and the KD‐NC group under 0, 0.2, 0.4, 0.8, 1.6, 3.2, 6.4 μM oxaliplatin treatment. GC, gastric cancer; KD‐KIF2A, knockdown‐kinesin family member 2A; KD‐NC, knockdown‐negative control; NS, non‐significance

In HGC‐27 cells, relative cell viability was decreased in the KD‐KIF2A group compared with the KD‐NC group under 800 μg/ml capecitabine treatment (*p* < 0.05) (Figure 4A) as well as under 0.4, 0.8, and 1.6 μM oxaliplatin treatment (all *p* < 0.05) (Figure 4B). Additionally, in NCI‐N87 cells, relative cell viability was reduced in the KD‐KIF2A group compared with the KD‐NC group under 1600 and 3200 μg/ml capecitabine treatment (both *p* < 0.05) (Figure 4C) as well as under 0.4 and 0.8 μM oxaliplatin treatment (both *p* < 0.05) (Figure 4D).

## 4 DISCUSSION

According to previous studies, KIF2A is highly expressed in the tissues of several kinds of tumors^20^, ^21^, ^22^: for example, a study shows that KIF2A expression is upregulated in the tumor tissue compared with adjacent tissue in NSCLC patients^20^; another study suggests that KIF2A expression in oral tongue cancer tissue is higher than that in paired adjacent tissue in SCCOT patients.^21^ In line with previous studies, our study discovered that KIF2A expression was elevated in the tumor tissue than in the adjacent tissue in GC patients. One explanation could be that: increased KIF2A reflected the higher proliferation speed of cells, and the proliferation speed of GC cells exceeded than that in the adjacent tissue cells. Therefore, KIF2A expression was elevated in tumor tissue than in adjacent tissue. Moreover, concerning the correlation of KIF2A expression with clinical features, previous studies suggest that elevated KIF2A expression associates with aggravating disease features in solid tumors.^15^, ^23^ For instance, elevated KIF2A expression correlates with bigger tumor size and higher clinical stage in osteosarcoma patients^15^; elevated KIF2A expression in tumor associates with larger tumor size and more advanced Barcelona clinic liver cancer stage in hepatocellular carcinoma patients.^23^ In our study, we found that increased KIF2A expression was associated with larger tumor size, more advanced N stage and TNM stage. The reason might be that: KIF2A promoted the proliferation, migration, and invasion of GC cancer cells through the AKT signaling pathway, thus facilitating the progression of GC and resulting in unfavorable disease features.^16^

Regarding the correlation of KIF2A with prognosis, previous studies have been performed to investigate its association with prognosis in various tumors.^13^, ^14^, ^17^, ^24^ For example, upregulated KIF2A independently associates with worse OS in facilitate lung adenocarcinoma patients^13^; EOC patients with overexpression of KIF2A has a shorter OS.^24^ Our study found that tumor KIF2A high was associated with poor accumulating OS in GC patients, which was similar to the findings of a previous study.^12^ Possible explanations could be that: (1) elevated KIF2A expression was associated with unfavorable disease features as mentioned above, which might indirectly cause shorter OS; (2) KIF2A might facilitate GC cell invasion by promoting membrane‐type 1 matrix metalloproteinase,^11^ which might subsequently influence progression after surgery and result in worse OS; (3) KIF2A might affect chemosensitivity after surgery and further influence OS.^25^ To further validate this possibility, we conducted a study on the effect of KIF2A on chemosensitivity of GC cells, which discovered that knockdown of KIF2A improved the chemosensitivity to oxaliplatin largely and only increased that to capecitabine mildly in GC cell lines. To the best of our knowledge, this was the first study to explore the effect of KIF2A on chemosensitivity of GC cell lines. However, there might exist bias caused by the CCK‐8 method. Moreover, in our study, we also found that no association was found in KIF2A with accumulating DFS, but KIF2A is an independent risk factor for worse DFS. The reason might be that: according to previous reports, GC had a low recurrence rate even after 10 years of surgical resection.^7^, ^26^ Thus, the number of patients suffering from recurrence is low, further causing low statistical power.

Although a lot of findings were identified in this study, there were still some limitations. Firstly, the sample size could be expanded to improve the statistical power. Secondly, this study did not investigate the molecular mechanism of KIF2A involved in GC progression. Thus in vivo and in vitro experiments could be further conducted. Thirdly, some confounding factors might affect findings, such as treatment of adjuvant chemotherapy or neoadjuvant chemotherapy in these patients. Thus, we adjusted the findings in multivariate Cox's regression model to eradicate the effect of these confounding factors on the prognostication effect of KIF2A in GC patients. Fourthly, the effect of KIF2A on other commonly used chemotherapeutic agents for GC such as S1 and paclitaxel, as well as the in‐depth molecular mechanism, could be further investigated. Fifthly, since most of the patients were Chinese, it was unclear whether the findings of this study were valid in patients from other regions.

In conclusion, KIF2A is highly expressed and its elevated expression associates with larger tumor size, advanced N stage, TNM stage as well as poor survival in GC patients; meanwhile, its knockdown enhances the chemosensitivity to oxaliplatin and capecitabine in GC cell lines. KIF2A might serve as a potential prognostic biomarker for improving the management of GC patients.

## CONFLICT OF INTEREST

The authors declare no competing interests.

## Data Availability

The datasets used and/or analyzed during the current study are available from the corresponding author on reasonable request.
